# Recent Advances in Laser-Ablative Synthesis of Bare Au and Si Nanoparticles and Assessment of Their Prospects for Tissue Engineering Applications

**DOI:** 10.3390/ijms19061563

**Published:** 2018-05-24

**Authors:** Ahmed Al-Kattan, Viraj P. Nirwan, Anton Popov, Yury V. Ryabchikov, Gleb Tselikov, Marc Sentis, Amir Fahmi, Andrei V. Kabashin

**Affiliations:** 1 Aix Marseille University, CNRS, LP3, 13288 Marseille, France; Viraj.nirwan@outlook.com (V.P.N.); popov@lp3.univ-mrs.fr (A.P.); ryabchikov@lp3.univ-mrs.fr (Y.V.R.); tselikov@lp3.univ-mrs.fr (G.T.); marc.sentis@univ-amu.fr (M.S.); kabashin@lp3.univ-mrs.fr (A.V.K.); 2 Faculty of Technology and Bionics, Rhin-waal University of Applied Science, Marie-Curie-Straβe 1, 47533 Kleve, Germany; Amir.Fahmi@hochschule-rhein-waal.de; 3 P.N. Lebedev Physical Institute of Russian Academy of Sciences, 53 Leninskii Prospekt, 199991 Moscow, Russia; 4 MEPhI, Institute of Engineering Physics for Biomedicine (PhysBio), 115409 Moscow, Russia

**Keywords:** laser ablation in liquid, Electrospinning, Nanoparticles, Nanofibers, Scaffolds, Tissue engineering, Nanotheranostics

## Abstract

Driven by surface cleanness and unique physical, optical and chemical properties, bare (ligand-free) laser-synthesized nanoparticles (NPs) are now in the focus of interest as promising materials for the development of advanced biomedical platforms related to biosensing, bioimaging and therapeutic drug delivery. We recently achieved significant progress in the synthesis of bare gold (Au) and silicon (Si) NPs and their testing in biomedical tasks, including cancer imaging and therapy, biofuel cells, etc. We also showed that these nanomaterials can be excellent candidates for tissue engineering applications. This review is aimed at the description of our recent progress in laser synthesis of bare Si and Au NPs and their testing as functional modules (additives) in innovative scaffold platforms intended for tissue engineering tasks.

## 1. Introduction

Tissue engineering is an important multidisciplinary field, which is focused on the development of biomaterial substitutes capable of replacing, detecting and treating failed or diseased tissues [1,2,3]. Due to a variety of tissues (e.g., bone, cartilage, nerve, cardiac/skeletal muscles, etc.), and their different functional and structural properties (e.g., stiffness, cell interconnection, etc.), the elaboration of substitutes presents a really challenging task, which has to gather a multitude of physicochemical characteristics (mechanical, electrical, etc.), structural properties (e.g., 3D architecture, surface topography, porosity, etc.) and advanced theranostic functionalities [4,5,6,7,8,9]. Biocompatibility and biodegradability are other key properties, which should be taken into account to ensure cell adhesion, growth and differentiation of surrounding tissues, preventing any rejection or toxicity issues [10,11,12]. 

Based on recent advances of nanotechnology, most efforts are now applied on the fabrication of nanostructured scaffolds, which could mimic the mesoporosity and nanoscale morphology of natural extracellular matrices (ECM) [13,14,15] and provide advanced functional properties for diagnostics/treatment of diseases or the restoration of biological functions [15,16]. A variety of scaffolds (hydrogel, nanofibers, etc.) made from ceramic, synthetic/natural polymers or composites are now explored for these tasks. Due to their chemical and structural similarity to natural mineral tissues, calcium phosphate ceramics present a class of tunable bioactive scaffolds, which are currently largely exploited in bone regeneration and orthopedic surgery [17]. Due to their capability of inducing osteoblastic differentiation, a plethora of coatings based on calcium phosphate compositions are employed as bioactive interfaces for implants [18,19]. However, clinical applications of these materials are still quite challenging because of brittleness, difficulty of shaping for implantation and uncontrolled degradation rate [20,21,22]. As another example, synthetic polymers (e.g., polystyrene) are also actively exploited as scaffolds to offer tunable architectures and degradability option [23]. Nevertheless, the interaction of many synthetic polymers with biological tissues is controversial because of their low bioactivity and side effects [23]. Owing to outstanding biocompatibility and biodegradability, natural polymers (chitosan, collagen, etc.) have appeared as very promising scaffolds, which can naturally promote cell adhesion and growth [24]. However, due to weak dissolution the shaping of natural polymers is challenging, while some of their physical properties including mechanical and conductivity characteristics must be improved [25,26]. 

One of promising ways to improve intrinsic properties of scaffolds and/or acquire additional functionalities consists in the incorporation of nanoparticles (NPs) as additives [27,28,29,30]. Considerable progress in this field has contributed to the fabrication of advanced multifunctional NPs for biomedical tasks including drug delivery, imaging and cell labeling. Applications of such NPs in tissue engineering could dramatically enhance physicochemical properties of scaffolds and contribute to their proper integration into tissue-specific microenvironments. Silver (Ag) NPs present a prominent example of non-exhaustive antibacterial objects, which are intensively exploited in tissue engineering tasks using a variety of polymeric scaffolds [31,32]. Iron-oxide NPs present another example of nanomaterials exhibiting antimicrobial and superparamagnetic properties, which can potentially be used in hyperthermia, gene therapy and bioimaging [33,34]. Owing to prominent optical and physical properties and chemical reactivity, gold (Au) NPs can offer many functionalities for biosensing, bioimaging and theranostics [28,29]. Due to their large surface area, porosity and biocompatibility, mesoporous silica (SiO_2_) NPs are also considered as relevant additives for drug delivery [35]. Finally, crystalline silicon (Si) looks as one of most promising candidates for tissue engineering, as it is ideally biocompatible [36] and biodegradable [37], as well can offer a large range of imaging and therapeutic functionalities, including room temperature photoluminescence for bioimaging [36,37,38,39,40], light-induced generation of singlet oxygen for photodynamic cancer therapy [41], and infrared radiation [42], radio frequency radiation [43] and ultrasound-induced [44] hyperthermia for cancer therapy. However, almost all currently employed NPs are synthesized by conventional chemical or electrochemical routes, which involve hazardous products (e.g., HF, nitrate salts, chloride, citrate, etc.) and various ligands. The presence of these products generally leads to surface contamination by residual toxic products, which is not consistent with targeted biomedical applications [45,46,47]. In addition, the elaboration of these nanomaterials takes place under extreme thermal, pH-metric and pressure conditions, which requires a rigorous control of the synthesis procedure. These reactions also frequently require different organic solvents (e.g., ethanol, THF, etc.) and many switching steps between organic/aqueous solutions, which complicates the fabrication and purification procedures [48,49]. 

Emerged as a new “green” nanosynthesis route, pulsed laser ablation gathers numerous benefits over chemical methods in the elaboration of ultraclean NPs [50]. This physical method implies the ablation of a solid target by pulsed laser radiation, which gives rise to a natural formation of nanoclusters [51,52]. If created in gaseous ambience, the nanoclusters can be deposited on a substrate forming a thin nanostructured film [53,54,55,56,57]. When created in liquid ambience, the nanoclusters can be released into the liquid forming nanoparticle colloidal solutions [58,59,60,61,62,63,64,65,66,67]. In both cases, the ablation can be performed in ultraclean environment (helium or argon gas, deionized water), excluding any contamination [50]. Here, the employment of ultra-short laser pulses can lead to the fabrication of extremely stable colloidal solutions of low-size-dispersed crystalline NPs even in the absence of any protective ligands [62,63,65]. In the case of Si, such procedure can be modified by the ablation (fragmentation) from microcolloids, prepared preliminarily by mechanical milling of a Si wafer [43,66,67]. Such a fragmentation method makes possible the fabrication of concentrated solutions of low size-dispersed Si-NPs with a variable mean size [66]. It was also shown that “bare” (ligand-free) surface can exhibit unusual reactivity and physicochemical properties [68,69,70,71]. Finally, bare laser-synthesized NPs are relevant nanotheranostics tools for biomedical applications [68,72]. We believe that due to the existence of such unique properties, the use of these nanomaterials as additives in tissue engineering looks very promising opening up opportunities for future innovative developments. 

This article reviews our recent achievements in the elaboration of bare inorganic and metallic NPs based on silicon and gold for biomedical applications, as well as highlights prospectives of such nanomaterials for tissue engineering applications. Here, we first remind the principle and the advantages of laser synthesis compared to conventional chemical synthesis routes. Second, the physicochemical characteristics of bare NPs and their interaction with biological matrices in vitro and in vivo are reviewed. We finally present results of our tests on the incorporation of Si and Au NPs as functional additives in electrospun nanofibers based on natural polymer chistosan-poly(ethylene oxide)(chitosan (PEO)) [73]. This multidisciplinary work aims to describe potential advantages of using bare NPs in tissue engineering applications. 

## 2. Pulsed Laser Ablation in Liquids (PLAL) for the Synthesis of Colloidal Nanomaterials

**Figure 1:**
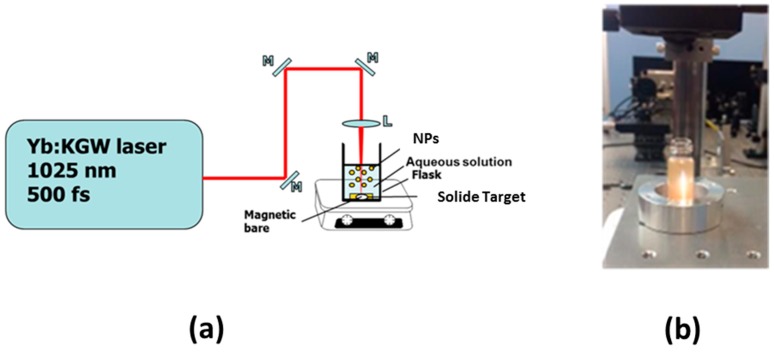
(**a**) Typical PLAL setup; (**b**) Illustrative image of colloidal Si NPs solution prepared by femtosecond (fs) laser fragmentation.

Driven by its flexibility, simplicity and rapidity, PLAL appears to be particularity important in the elaboration of ultra-clean, bare (ligand-free) NPs for a variety of applications, including electronics, energy production and biomedicine (for review, see [50,74]. Based on a combination of top-down and bottom-up approaches, this method profits from laser-target interaction to ablate material of the target and thus naturally form nanoclusters. The nanoclusters then coalesce in liquid medium to produce a colloidal NPs solution. Figure 1 illustrates a basic experimental set-up, which offers an easy handling to produce colloidal solutions of nanomaterials. In conventional ablation geometry, the set-up consists of a pulsed laser and focusing optics to ablate a target placed on the bottom of a cuvette filled with a liquid solution (Figure 1a). In this case, the platform with the target should be constantly moved to avoid the ablation from the same area on the target. In an alternative fragmentation setup, material is ablated from liquid-dispersed micro/nano particles, while the liquid is constantly mixed by a magnetic stirrer (Figure 1b). 

PLAL has numerous advantages over conventional chemical methods such as the possibility for NPs synthesis in ultrapure non-contaminated solutions (e.g., deionized water) without surfactants or ligands. NPs formed under these conditions can exhibit unique “bare” surface, which is characterized by different reactivity compared to chemically-synthesized NPs [69,70,71,72,73]. It is also important that such ultrapure surface does not require any additional purification, as it often takes place in the case of chemically-synthesized NPs. Here, to condition an appropriate chemical composition ranging from elemental to hydroxide, water can be replaced by organic solvents, polymer or saline solutions. Moreover, by varying the liquid composition (e.g., oils, superfluids, etc.) one can monitor the NPs shape [74]. As another advantage, laser-ablative synthesis makes possible (bio)functionalization of formed nanomaterials in situ [74]. 

It should be noted that laser ablation mechanism is a complex process involving extreme phenomena (shock wave, plasma plume and cavitation bubble, etc.) under severe physical and thermodynamic conditions (thousands of kelvins and hundreds of pascals). Furthermore, all processes take place simultaneously during a very short time period (a few ns) and depend on laser parameters (pulses length, wavelength, fluence, etc.) [75]. Many experimental methods (spectroscopic analysis, acoustic measurements, x-ray imaging techniques, acoustic measurements, CCD cameras observations) and theoretical modeling investigations were devoted to clarifying the ablation mechanism [75]. In most cases, laser ablation tends to provide a broadened size dispersion of NPs (from several tens to hundreds of nm) with polymodal size populations. As a debated scenario, a sequence of different ablation mechanisms occurs during plasma plume expansion (cooling) and the explosion of a cavitation bubble. Such phenomena are especially important for a “long” ns laser ablation regime. The addition of reactive chemical products during the ablation process makes possible efficient “chemical” control of NPs size, but in this case the cleanness of NPs can be compromised. On the other hand, laser ablation at ultrashort regime (fs) looks like the most promising “fine” tool, which is now accepted by the whole laser processing community. Due its specific conditions, much less radiation is transferred to the cavitation bubble, which limits cavitation phenomena and thus makes possible much more rigorous control of NPs size and size dispersion [50,75]. 

**Figure 2:**
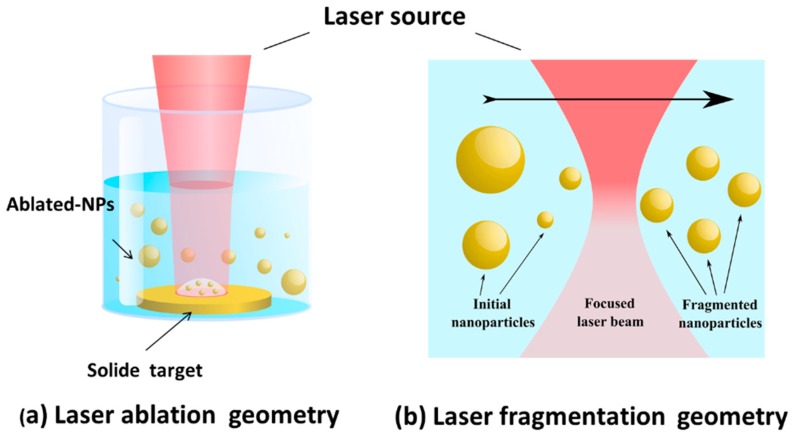
Schematic presentation of laser ablation (**a**) and laser fragmentation (**b**) geometries.

It is important that initial colloidal NPs solutions obtained by laser ablation (Figure 2a) can be subjected to the second laser “fragmentation” step (Figure 2b) [65]. The fragmentation mechanism still not fully understood, but “photothermal evaporation” and “Coulomb explosion” are considered as the main mechanisms responsible for ablation. Moreover, preliminary colloids can be directly obtained from micropowder suspensions [66,68]. Here, fs laser ablation from micro/nano colloids manifested itself a very efficient method to achieve desired controllable size characteristics of formed NPs. In general, PLAL looks as a reliable tool for the fabrication of a variety of nanomaterials, which enables one to control NPs characteristics by adjusting laser parameters (focusing point position, fluence, repetition rate, fragmentation duration, etc.) and physicochemical conditions (e.g., concentration of raw material).

## 3. PLAL Synthesis of Bare Nanomaterials for Biomedical Applications

### 3.1. Bare Laser-Synthesized Si Nanoparticles 

**Figure 3:**
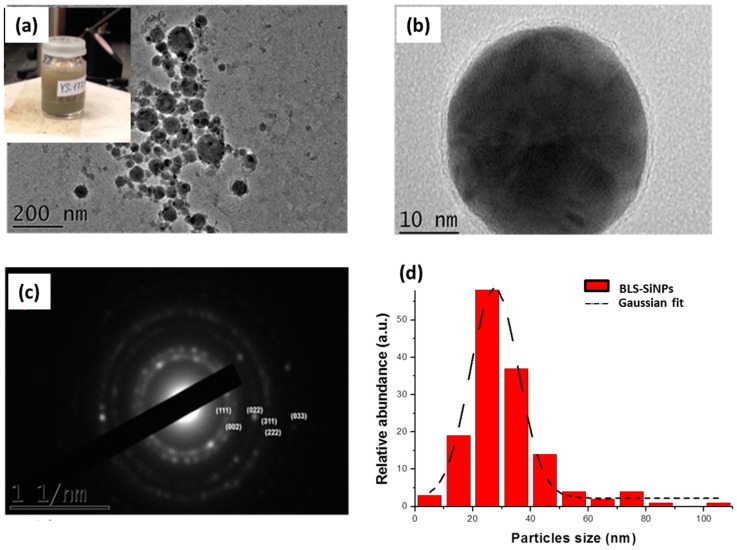
(**a**) HR-TEM image of Si NPs obtained by laser fragmentation at 0.35 g·L^−1^ initial concentration of microcolloids (Inset, typical image of Si NPs solution). (**b**) Single laser-synthesized Si nanoparticle. Characteristic electron diffraction pattern of Si NPs (**c**) and corresponding size distributions (**d**). Adapted from ref. [67].

Silicon (Si) is a group IV semiconductor, which participates in many biochemical processes, including bone mineralization (e.g., osteoblastogenesis), connective tissue metabolism, signal transduction [76,77,78]. Moreover, Si improves the adsorption of crucial minerals such as magnesium and copper, which are involved in the proliferation of lymphocyte cells and their immune response. In addition, Si nanostructures are water-dissolvable and biodegradable, as in biological environment they convert into orthosilicic acid Si(OH)_4_, which is naturally excreted with urine [37]. Finally, Si NPs offer a large panel of applications in biomedicine including photoluminescence-based imaging [39,40,79], photodynamic therapy and hyperthermia-based therapy for cancer treatment [41,42,43,44]. Si NPs can be synthesized using chemical reduction methods, microemulsion techniques, electrochemical synthesis, etc., which typically require numerous purification steps to clean the NPs surface [37,47,80,81,82]. Here, specific installations (hood, vacuum box, etc.) and operating skills are required. We recently introduced ultrashort (femtosecond) laser ablation in aqueous solutions as a novel approach to fabricate ultrapure Si NPs for biomedical applications [66,67]. In a typical laser-synthesized protocol, Si NPs are prepared from Si microparticles powder dispersed at 0.35 g·L^−1^ in deionized water by sonication step for 30 min. The microparticles are fragmented by focused femtosecond laser irradiation (Yb:KGW laser, Amplitude systems, 1025 nm, 480 fs, 1 kHz) for 1 h (for more details see ref. [67]). Physicochemical characterization showed that so formed Si NPs have a tunable mean side between 10 and 100 nm under narrow size dispersion. Structural and analytical measurements showed that the NPs are surrounded by a thin oxidized layer of SiO_x_ (1 ≤ x ≤ 2) with a ζ-potential of −45 ± 1.5 mV preventing thus any aggregation phenomenon between the Si NPs (Figure 3b). 

**Figure 4:**
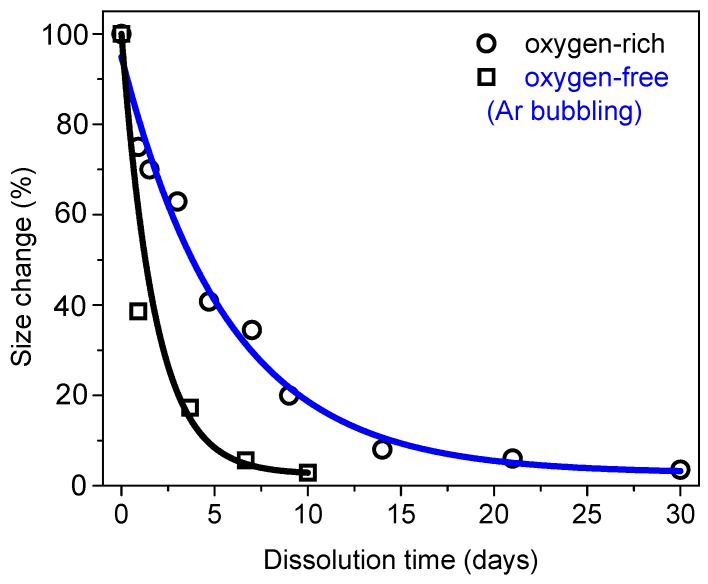
Size evolution (in percent, relative to the initial size of Si NPs prepared under oxygen-rich (black) and oxygen-free (blue, Ar bubbling) conditions as a function of dialysis duration in deionized water. Adapted from ref. [67].

Moreover, we established that by varying the amount of dissolved oxygen in water, one can control the oxidation state and potentially create silicon oxide defects inside Si NPs crystals, which can lead to much accelerated dissolution of NPs in aqueous solutions (Figure 4) [67]. Other advantages of PLAL approach are related to the possibility of controlling mean NPs size by varying the initial Si microparticles concentration [66]. Such approach can provide “calibrated” additives with monitored structural properties.

**Figure 5:**
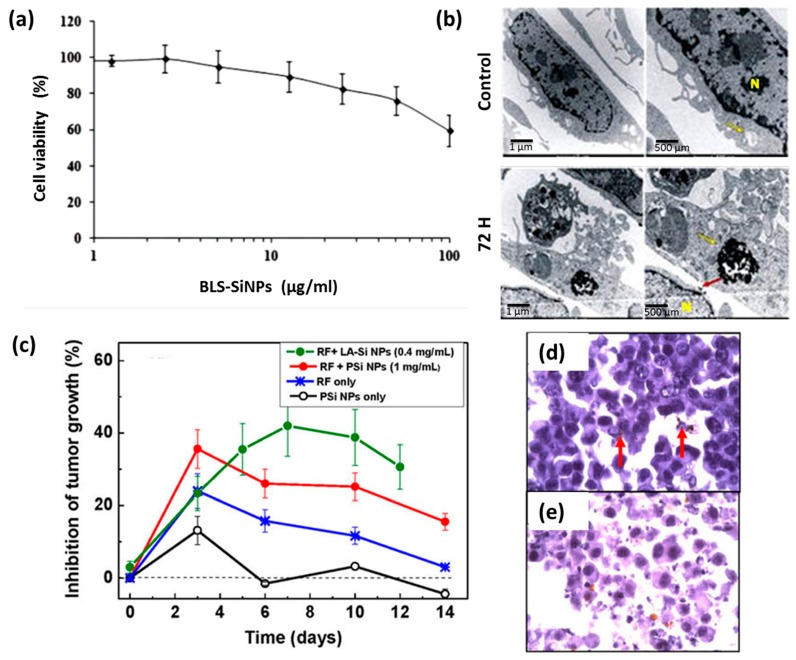
(**a**) MTT assays of HMEC cells viability following their exposure to different concentration of Si NPs (1.25–100 µg/mL) for 72 h. (**b**) TEM images of HMEC cells showing kinetics of Si NPs internalization 72 h after incubation time with 50 µg/mL of NPs. (**c**) Inhibition of the tumor growth after the following treatments: the injection of Si NPs suspension without RF irradiation (black curve); 2 min treatment of tumor area by RF irradiation with the intensity of 2 W/cm^2^ (blue); injection of a suspension of porous Si NPs (PSi NPs) (0.5 mL, 1 mg/mL) followed by 2 min RF irradiation treatment (red); injection of a suspension of laser-synthesized Si NPs (LA-Si NPs) (0.2 mL, 0.4 mg/mL) followed by 2 min RF irradiation treatment (green). (**d**,**e**) are histology images of a tumor area 1 h and 3 days after the PSi NP injection and RF-based treatment using PSi NPs as nanosensitizers, respectively. Cancer cells are visible as dark blue spots. Examples of agglomerations of PSi NPs in the cells are indicated by red arrows. Adapted from refs. [43,83].

The interaction of Si NPs with biological matrices was investigated in vitro and in vivo in our earlier studies [67,83]. No obvious cytotoxicity effect on human cells (HMEC) was observed up to 100 µg·mL^−1^ with cell survival rate around 80% (Figure 5a). In addition, TEM examination revealed that Si NPs are readily uptaken by cells via classical endocytosis mechanism without damage of cell compartments (Figure 5b). In vitro study was also completed by in vivo tests performed in a nude mouse model at different incubation time (from 3 h to 7 days) with a single dose (20 mg/kg) of intravenous administration [83]. Based on the examination of behavior of mice and their growth, we concluded that all animals showed normal physiological activities without lethargy or apathy. The biodistribution and the fate of Si NPs were followed by a control of a panel of biochemical parameters and the examination of organ tissues. This study revealed that Si NPs were completely safe. Furthermore, these NPs were cleared from biological matrices within one week, while similar porous Si-based nanoformulations prepared by electrochemical routes require 4–6 weeks for the excretion [37]. The functionality of laser-synthesized Si NPs as sensitizers of radiofrequency (RF)-induced hyperthermia was tested on Lewis lung carcinoma in vivo and compared to porous silicon-based nanostructures (Figure 5c–e) [43]. Here, we observed efficient tumor inhibition without any side effects, while laser-synthesized NPs demonstrated much stronger therapeutic outcome. We believe that such sensitizing properties of laser-synthesized Si NPs can be used as a novel functionality in the development of tissue engineering platforms. 

### 3.2. Bare Laser-Synthesized Au Nanoparticles 

Nanostructured gold (Au) has attracted a considerable attention of biomedical community due to their unique physical, optical and chemical properties [84,85]. Owing to optical excitations of free electron oscillations (plasmons), electric field is strongly enhanced in the vicinity of metal surface, which can be used in various applications, including biosensing [86,87,88,89], imaging [90,91], photothermal therapy [92,93,94], gene and drug delivery. Numerous methods have been reported to fabricate a wide variety of Au NPs shapes (nanospheres, nanorods, nanoplates, nanoshells, etc.) opening a wide avenue for applications in energy, biomedicine, material science and tissue engineering. Here, the surface of Au NPs can be functionalized by polymers (e.g., PEG) [95,96], functional groups (e.g., Amine and Carboxyl) [97,98], as well as by biomolecules including DNA [99] and peptides [100]. Such functionalizations can help to enhance specificity and efficacy of Au NPs toward specific cell types and organelles such as nucleus and mitochondria. However, in general such NPs are fabricated by chemical routes, involving stabilizing molecules or ligands, which are not always biocompatible [101]. First, the presence of stabilizing agent on Au NPs surface can potentially hinder their direct interactions with biological environment and can compromise their future functionalization [102,103]. Second, these molecules can interfere with plasmonic properties of Au NPs [104]. 

**Figure 6:**
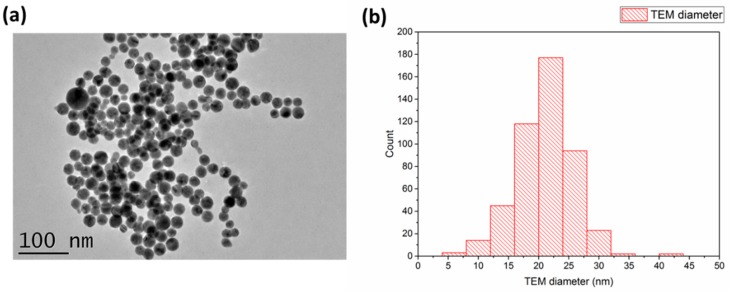
Typical HR-TEM image of Au NPs prepared by PLAL (**a**) and corresponding size distribution (**b**).

To overcome such limitations, we recently elaborated PLAL technique to synthesize bare Au NPs in aqueous solutions in the absence of any stabilizing molecules (Figure 6) [62,63,65,68]. In a typical procedure a solid Au target (99.99%, GoodFellow, France) was placed at the bottom of the glace vessel and filled with 7 mL of deionized water. The target was then irradiated with femtosecond laser (Yb:KGW laser, Amplitude systems, 1025 nm, 480 fs, 1 kHz) for 15 min. In order to reduce size dispersion, Au NPs produced by laser ablation step were then subjected to the second “fragmentation” step for 30 min (for more details see ref. [72]). Structural and microscopic observations revealed that Au NPs were spherical in shape and free from any residual contaminants, enabling high chemical [69,70,71] and catalytic [68] activity. In addition, due to the partial oxidation of surface (Au–O^−^/Au–OH^−^), the Au NPs exhibit a negative surface charge (−23 ± 2.3 mV) conferring thus a great stability and limiting any agglomeration effect. The interaction of such NPs with biological matrices was assessed during in vitro tests under relatively high concentration (10 mg/L^−1^) of NPs up to 72 h. TEM analyses demonstrated biological safety characteristics of Au NPs without any side effects on morphology and cytoskeleton cells [72]. Au NPs were internalized by a classical endocytosis mechanism without penetration into the nucleus cell. In addition, the analysis of protein corona on NPs surface revealed interactions with abundant proteins such as Albumine and Apos, which are known to play crucial roles in intracellular trafficking [72]. Due to their unique structures and excellent biocompatibility, bare Au NPs can be considered alternative candidates as additives for tissue engineering. 

## 4. Potential Applications of BLS-NPs in Tissue Engineering 

Biological tissue presents a complex environment with specific structural, biological, chemical and physical characteristics. Therefore, the creation of artificial functional tissue structures (scaffolds) has to gather variety of properties such as a good cell adhesion, high porosity, adequate pore size for cell seeding and diffusion, structural rigidity, biocompatibility and biodegradability. Many studies are now devoted to the elaboration of functional scaffolds, which could mimic the ECM. Scaffolds can be fabricated by variety conventional techniques (e.g., solvent casting/particle leaching [105]), but electrospinning looks as the most promising approach to fabricate biocompatible/biodegradable nanofibrous scaffolds. Based on the application of electrical field in polymer solutions, this process offers plenty of advantages such as the possibility of working with a variety of materials including natural/synthetic polymers and their composites, generation of micro- to nano-scale nanofibers, cost effectiveness and easy scaling-up [106,107]. However, despite these benefits and approach flexibility, a number of problems need to be solved such as the fabrication of uniform nanofibers with desired diameter, morphology, mechanical strength, conductivity and chemistry. On the other hand, the elaboration of electrospun nanofibers with multi-functionalities (biological and therapeutic characteristics) is still required. 

We recently carried out tests to explore the potential of using bare laser-synthesized Si and au NPs as functional additives in order to (i) improve/optimize intrinsic properties of nanofibers; (ii) enable advanced biomedical/biological properties. As a first approach, we recently functionalized biologically-derived polymer based on nanofibers chitosan (PEO) by bare Si and Au NPs [73]. At optimized chitosan:PEO ratio, the NPs were directly introduced at increased concentration in the polymer solution before electrospinning. Numerous analyses were then conducted on obtained nanofibers based on microscopic, thermal and analytical methods. First, it appeared that the NPs were properly attached via electrostatic interaction and homogenously dispersed on the nanofiber surface, while the presence of NPs did not affect the morphology of fiber networks and their chemical properties. Second, we observed a reduction of the fiber diameter by a factor 2 when the fibers are co-electrospun with Si NPs. In addition, functionalized nanofibers exhibited better thermal stability at higher temperature and this effect was especially prominent for Si NPs. Safety properties of the hybrid scaffold were also assessed by preliminary MTT tests and did not show ant toxicity. 

**Figure 7:**
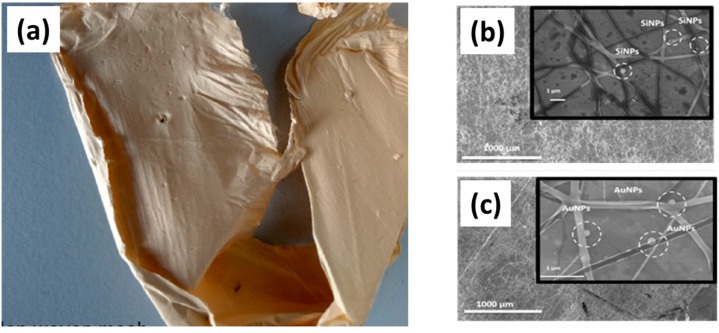
(**a**) Illustrative image of electrospun chitosan(PEO) nanofibers functionalized with bare Si NPs. (**b**) SEM of hybrid chitosan (PEO) nanofibers functionalized with bare Si NPs at 30 wt. %. (**c**) SEM of hybrid chitosan (PEO) nanofibers functionalized with bare Au NPs at 30 wt. %. Adapted from ref. [73].

These first tests confirmed the possibility of using NPs as functional additives in the elaboration of innovative scaffolds for tissue engineering (Figure 7). In particular, the presence of NPs on the nanofibers surface can be exploited as additional anchoring site interacting with cells. Here, the bare surface of laser-synthesized NPs looks very important as it can be tuned with specific biomolecules and growth factors to increase the nanofibers bioactivity toward cells. On the other hand, NPs can be used as sensitive probes to track variety of biomolecules (DNA, RNA, protein, etc.) and other materials including metal ions. Furthermore, the reduction of nanofibers diameter can potentially lead to higher bioactivity characteristics as it was noted in literature [108]. Besides, the incorporation of BLS-NPs into nanofibers improves thermal stability of the fiber matrix, which can be exploited for therapeutic applications and extended to other physicochemical parameters such as pH control. Despite encouraging results, the development of NPs as functional additives is in its early stage and many issues should still be clarified. Here, many physical properties including mechanical and electrical characteristics have to be assessed. In addition, the scaffold fate (e.g., dissolution of nanofibers, release of NPs from scaffold, etc.) has to be monitored and evaluated. Other parameter such as the size of NPs has to be varied to highlight their effect on the physicochemical properties of the fibers. 

## 5. Conclusions

In conclusion, bare laser-synthesized NPs open a wide range of opportunities toward the elaboration of functional scaffolds for tissue engineering enabling advanced biomedical modalities. First, PLAL method enables to fabricate NPs exempt of any contaminants, while the NPs surface can exhibit high reactivity and much better biocompatibility compared to chemically-synthesized Si and Au counterparts. Second, structural properties of laser-synthesized NPs can be easily designed to control their size and dissolution behavior. Third, one can use Si and Au NPs to enable a variety of therapy modalities [109], as well as imaging modalities including fluorescence imaging [40], SERS [110], SEIRAS [111]. As first preliminary work, we highlighted the possibility of incorporating of Si and Au NPs as functional additives for hybrid-electrospun nanofibers based on chitosan (PEO), without any effect on nanofiber compositions. Here, we observed a drastic decrease of nanofiber diameter promising a much improved bioactivity of nanofibers, while thermal analysis revealed a better stability of nanofibers at higher temperatures which can be exploited for advanced therapeutic tasks. Finally, the presence of NPs on the nanofibers promises its additional reactive surface toward biological tissue. Si and Au NPs can offer the opportunity to fabricate innovative scaffolds systems, which are capable of treating specific information related to surrounding tissues. The employment of NPs as functional modules for tissue engineering is still in a very early stage. Intensive research is still required to assess all potential benefits from such nano-engineered systems. 

## Abbreviations

NPs	Nanoparticles
PLAL	Pulsed Laser Ablation in Liquid
